# Increased retinal venular calibre in acute infections

**DOI:** 10.1038/s41598-021-96749-y

**Published:** 2021-08-26

**Authors:** Cara Fitt, Thao Vi Luong, Damian Cresp, Anastasia Hutchinson, Karen Lim, Lauren Hodgson, Deb Colville, Judy Savige

**Affiliations:** 1 Department of Medicine, Melbourne Health and Northern Health, Royal Melbourne Hospital, The University of Melbourne, Parkville, VIC 3050 Australia; 2 Northern Health, Epping, VIC 3076 Australia; 3 Department of Ophthalmology, Royal Victorian Eye and Ear Hospital, The University of Melbourne, East Melbourne, VIC 3010 Australia

**Keywords:** Biomarkers, Cardiology, Risk factors

## Abstract

Population-based studies have demonstrated that increased retinal venular calibre is a risk factor for cardiac disease, cardiac events and stroke. Venular dilatation also occurs with diabetes, obesity, dyslipidemia and autoimmune disease where it is attributed to inflammation. This study examined whether the inflammation associated with infections also affected microvascular calibre. Participants with infections and CRP levels  >  100 mg/L were recruited from the medical wards of a teaching hospital and assisted to complete a demographic and vascular risk factor questionnaire, and to undergo non-mydriatic retinal photography (Canon CR5-45NM, Japan). They were then treated with appropriate antibiotics, and underwent repeat retinal imaging when their CRP levels had fallen to less than 100 mg/L. Retinal images were examined for arteriole and venular calibre using validated semi-automated software based on Knudtson’s modification of the Parr-Hubbard formula (IVAN, U Wisconsin). Differences in inflammatory markers and calibre were examined using the paired t-test for continuous variables. Determinants of calibre were calculated from multiple linear regression analysis. Forty-one participants with respiratory (27, 66%), urinary (6, 15%), skin (5, 12%), or miscellaneous (3, 7%) infections were studied. After antibiotic treatment, participants’ mean CRP levels fell from 172.9 ± 68.4 mg/L to 42.2 ± 28.2 mg/L (p < 0.0001) and mean neutrophil counts fell from 9 ± 4 × 10^9^/L to 6 ± 3 × 10^9^/L (p < 0.0001). The participants’ mean venular calibre (CRVE) decreased from 240.9 ± 26.9 MU to 233.4 ± 23.5 MU (p = 0.0017) but arteriolar calibre (CRAE) was unchanged (156.9 ± 15.2 MU and 156.2 ± 16.0 MU, p = 0.84). Thirteen additional participants with infections had a CRP > 100 mg/L that persisted at review (199.2 ± 59.0 and 159.4 ± 40.7 mg/L, p = 0.055). Their CRAE and CRVE were not different before and after antibiotic treatment (p = 0.96, p = 0.78). Hospital inpatients with severe infections had retinal venular calibre that decreased as their infections resolved and CRP levels fell after antibiotic treatment. The changes in venular calibre with intercurrent infections may confound retinal vascular assessments of, for example, blood pressure control and cardiac risk.

## Introduction

Examination of the retinal small vessels enables direct visualisation of structural and pathologic features of the microcirculation during life. Altered vessel calibre reflects the earliest sign of microvascular damage from systemic disease. Population-based studies suggest that retinal small vessel calibre predicts an increased risk of cardiac disease, cardiac events and stroke^1^. Arteriolar narrowing in men and venular dilatation in women correlate with this increased risk^1^. Coronary angiographic studies have found that calibre is related to more severe coronary disease and some types of intracoronary plaque^2,3^. Retinal microvascular disease also correlates with diastolic heart failure and progressive renal impairment^2,4^.

However microvascular calibre has multiple determinants. Arteriole calibre is smaller in men, with increased age, renal failure and poorly controlled hypertension^5^. Venular calibre is larger with smoking, diabetes, obesity, and dyslipidemia^6–9^. Venular calibre is also larger in chronic inflammatory diseases such as chronic obstructive pulmonary disease^10^, rheumatoid arthritis and SLE^11^. Calibre is not fixed but varies with physiological activity, for example, after haemodialysis^12^. The effect of acute infections on microvascular calibre has not been studied previously.

C reactive protein (CRP) is an acute phase reactant that is elevated in infections. It is synthesized by the hepatocytes in response to increased IL-6^13^, and levels increase within 6 h of disease onset, and start to fall within 48 h of the response to antibiotic treatment^14^. CRP was previously considered a bystander in the inflammatory response, but recent evidence suggests that it is an inflammatory mediator that is inversely proportional to basal endothelial nitric oxide synthesis^15,16^, and hence potentially directly involved in endothelial dysfunction^17,18^ and vascular injury^19^. CRP also represents a non-traditional risk factor for cardiac disease itself^20^, since elevated CRP levels are an independent cardiovascular risk factor^21^ that is associated with a poorer clinical outcome^22^.

This study examined the effect of acute infections on retinal small vessel calibre because any changes might interfere with the ability to use calibre to assess blood pressure control or predict cardiac risk in individuals with coincidental infections.

## Subjects and methods

### Study design

This was a single centre paired observational study of individuals with infections who underwent assessment for retinal microvascular calibre before and after antibiotic treatment.

Consecutive individuals admitted over a 6 month period to a general medical ward with an infection and CRP level > 100 mg/L were invited to participate. Participants were assisted to complete a structured questionnaire that included demographic and medical details, as well as vascular risk factors (hypertension, diabetes, dyslipidemia, smoking history) and medications. Participants continued with their pre-existing treatment for hypertension and diabetes but were not allowed to smoke while in hospital. Relevant laboratory test results (CRP, FBE, neutrophil counts, albumin, eGFR) were obtained from their electronic medical records, and participants underwent non-mydriatic retinal photography. They were treated with antibiotics, and those whose CRP levels fell to levels < 100 mg/L underwent repeat retinal photography. Retinal images were then examined for arteriole and venular calibre at a grading centre by a trained grader.

Inclusion criteria were age ≥ 18 years, infections with an initial CRP level > 100 mg/L, and CRP < 100 mg/L after treatment. Exclusion criteria were bilateral ungradable retinal images. Data were also available for some individuals where the second CRP level was still > 100 mg/L.

The study was approved by the Human Research Ethics Committee at Northern Health, according to the Principles of the Declaration of Helsinki, and all participants provided written, informed consent.

### Retinal imaging and vessel calibre measurement

Digital retinal imaging was performed using a non-mydriatic retinal camera (Canon CR5-45, Tokyo). At least 2 standardised 45° colour digital images were taken of each eye, with one centred on the optic disc and the other on the macula. In general the right retina was examined on both occasions, but if this were ungradeable, the left was used.

Retinal arteriole and venular calibre were measured by a grader masked to subject identity and treatment, using a computer-assisted semi-automated imaging software (IVAN, University of Wisconsin) and a standardised protocol at the Centre for Eye Research Australia^23,24^. This identified the six largest arterioles and venules in a ring 0.5–1.0 disc diameters from the optic disc margin, and the Central Retinal Arteriole (CRAE) and Venular Equivalents (CRVE) were then determined from Knudtson’s revision of the Parr-Hubbard formula. Grading automatically took into account axial length. Fractals and tortuosity were not assessed because these were unlikely to change over the short period of follow-up. This grading method was highly reproducible in the laboratory with intra-grader coefficients of variation of 0.986 and 0.989 for CRAE and CRVE respectively^11^.

### Statistical analysis

Differences in clinical and laboratory characteristics in individual subjects were compared using the paired t-test for continuous variables. The contributions of inflammatory and vascular risk factors to small vessel calibre were examined using univariate analysis and independent determinants from multivariate analysis (SPSS21.0). This was a pilot study and it was not possible to perform a power calculation since the effect of infections on calibre was not known. The aim was to generate hypotheses and no correction was performed for multiple analyses.

## Results

### Participant characteristics

Forty-one individuals fulfilled the conditions of recruitment where their initial CRP was > 100 mg/L, the follow up CRP was < 100 mg/L and where they had at least one gradable retinal image. The study cohort comprised 29 men (71%) and 12 women (29%) with a mean age of 65.7 ± 18.4 years (Table 1). Seventeen (41%) had hypertension, and the overall mean arterial pressure was 86 ± 12 mm Hg. Thirteen (32%) had diabetes, 9 had dyslipidemia (22%), and 31 (76%) were current or former smokers. Their mean eGFR was 65 ± 26 mL/min/1.73 m^2^. Study participants had infections of the respiratory tract (n = 27, 66%), urinary tract (6, 15%), skin (n = 5, 12%), or endocarditis (n = 3, 7%). They were treated with ceftriaxone (for respiratory, urinary tract infections), flucloxacillin (cellulitis) or ampicillin and gentamicin (endocarditis).

**Table 1:** Participant characteristics where 1st CRP > 100 and 2nd CRP < 100 mg/L.

Characteristic (n = 41)	
Age (mean ± SD, years)	65.7 ± 18.4
Gender (male, %)	29 (71%)
Hypertension (≥ 140/90 mm Hg, %)	17 (41%)
Mean arterial pressure (mean ± SD, mmHg)	86 ± 12
Treated with antihypertensives (%)	15 (37%)
Diabetes (%)	13 (32%)
Dyslipidaemia (%)	9 (22%)
Current or former smoker (%)	31 (76%)
**Infection**	
Upper respiratory tract	27 (66%)
Urinary tract	6 (15%)
Cellulitis, endocarditis (n = 2)	8 (19%)

All participants had their second retinal photograph taken within 5 days of the first. Participants with hypertension continued their treatment unchanged during the study. Their commonest medications were angiotensin receptor blockers or angiotensin converting enzyme inhibitors together with a calcium channel blocker where a second agent was required. Likewise participants with diabetes continued routine treatment. None was treated with a vasoconstrictive agent during the treatment period.

### Measures of inflammation

**Figure 1:**
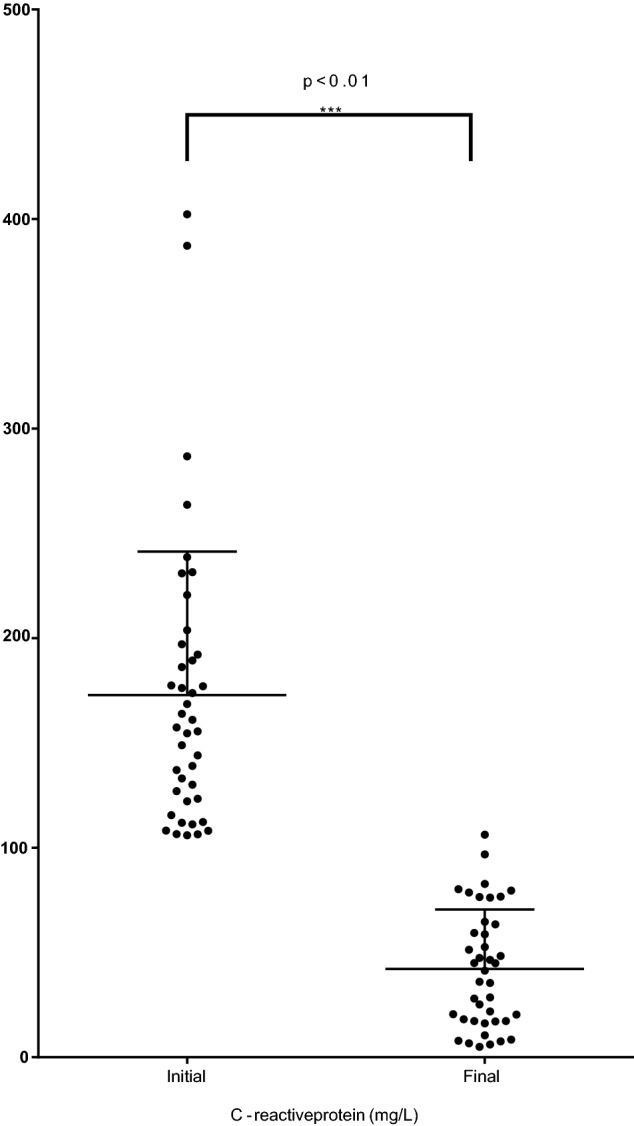
Initial and final CRP levels.

**Figure 2:**
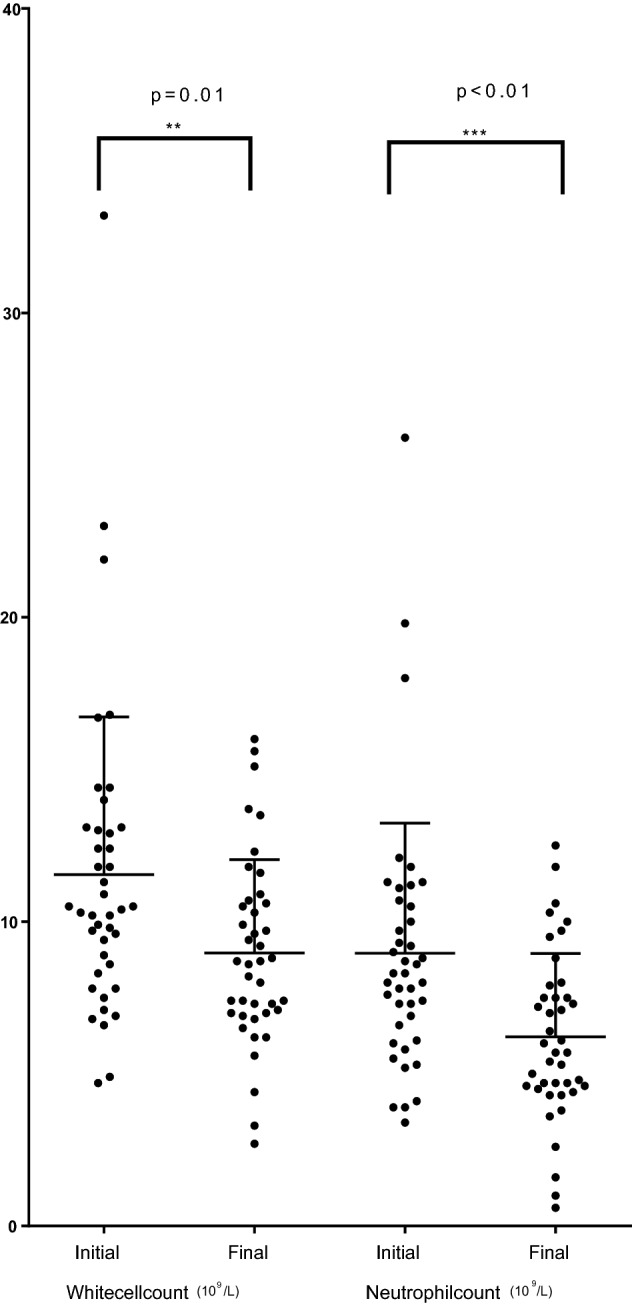
Initial and final total WBC and neutrophil counts.

The participants’ mean CRP level at recruitment was 172.9 ± 68.4 (range 106.4 to 402.3) mg/dL which fell after treatment to 42.2 ± 28.2 (range 7.6 to 96.8) mg/L (diff 130.6, 109.4 to 151.9, p < 0.0001) (Table 2). Their mean white cell count of 12 ± 5 × 10^9^/L fell to 9 ± 3 × 10^9^/L (diff 2.6, 1.4 to 3.6, p < 0.0001), and their mean neutrophil count of 9 ± 4 × 10^9^/L fell to 6 ± 3 × 10^9^/L (diff 2.5, 1.4 to 3.6, p < 0.0001) (Figs. 1, 2). Hb, serum albumin and eGFR levels were not different before and after antibiotic treatment.

**Table 2:** Characteristics at recruitment and after CRP levels fell to < 100 mg/L.

Characteristic	Pre-treatment (mean ± SD)	Post-treatment (mean ± SD)	*Difference, 95% CI	*p-value
White cell count (10^9^/L)	12 ± 5	9 ± 3	2.6 (2.1 to 3.2)	** < 0.0001**
Neutrophil count (10^9^/L)	9 ± 4	6 ± 3	2.5 (1.4 to 3.6)	** < 0.0001**
CRP (mg/L)	172.9 ± 68.4	42.2 ± 28.2	130.6 (109.4 to 151.9)	** < 0.0001**
Serum albumin (g/L)	30 ± 5	30 ± 5	− 0.15 (− 1.77 to 1.46)	0.84
Hemoglobin (g/L)	124 ± 19	125 ± 16	− 0.6 (− 4.5 to 3.4)	0.77
eGFR (mL/min/1.73m^2^)	65 ± 26	67 ± 25	− 3.8 (− 10.2 to 2.6)	0.22
Central retinal arteriole equivalent calibre (MU)	156.9 ± 15.2	156.2 ± 16	0.27 (− 2.5 to 3.0)	0.84
Central retinal venular equivalent calibre (MU)	240.9 ± 26.9	233.4 ± 23.5	8.8 (3.5 to 14.1)	**0.0017**

Results in bold where p < 0.05.

*CRP* C-reactive protein.

*By two-tailed paired t test.

### Retinal vessel calibre

The participants’ mean arteriole calibre (CRAE, Central Retinal Arteriole Equivalent) was not different at recruitment and after treatment (156.9 ± 15.2 MU and 156.2 ± 16.0 MU respectively, diff 0.3, -2.5 to 3.0, p = 0.84) (Table 2).

However their mean venular calibre decreased from 240.9 ± 26.9 MU to 233.4 ± 23.5 MU (diff 8.8, 3.5 to 14.1 p = 0.0017) (Table 2).

The change in venular calibre correlated with initial white cell and neutrophil counts (p both < 0.01), but not with initial CRP, serum albumin, gender, hypertension, diabetes, smoking history dyslipidaemia, haemoglobin level or renal function (p all NS). The initial white cell count was the most significant determinant of increased venular calibre after multivariate stepwise regression (coefficient = 1.30, 95% CI 0.38 to 2.21, p < 0.01) (Table 3).

**Table 3:** Determinants of change of retinal venular calibre in cohort where final CRP < 100 mg/L.

Variable	Coefficient	95% CI		p-value
Age	Excluded variables			0.32
Gender				0.31
Diabetes				0.92
Dyslipidemia				0.57
Hypertension				0.70
Smoking history				0.59
Initial white cell count	1.30	− 0.38	2.21	** < 0.01**
Initial neutrophil count	Excluded variables			0.89
Initial CRP (mg/L)				0.39

Results in bold where p < 0.05.

### Participants with follow-up CRP > 100 mg/L

Data were available for 13 further individuals with infections whose initial and follow-up CRP levels were both > 100 mg/L (means, SD, 199.2 ± 59.0 and 159.4 ± 40.7 mg/L respectively, (p = 0.055) (Tables 4 and 5). Their mean age was 55 ± 10 years, 10 were male (77%), 8 (62%) had hypertension, 2 had diabetes (15%) and one was a smoker (7%). Four had an upper respiratory tract (31%), two had a urinary tract (16%) and 7 (50%) had other infections (cellulitis, endocarditis, cholecystitis). Their changes in CRAE and CRVE after treatment were not significant (p = 0.96, p = 0.78 respectively).

**Table 4:** Participant characteristics where initial and final CRP levels were both > 100 mg/L.

Characteristic (n = 13)	
Age (mean ± SD, years)	55 ± 10
Gender (male, %)	10 (77%)
Hypertension (≥ 140/90 mm Hg, %)	8 (62%)
Mean arterial pressure (mean ± SD, mmHg)	84 ± 10
Treated with antihypertensives (%)	8 (62%)
Diabetes (%)	2 (15%)
Dyslipidaemia (%)	2 (15%)
Current or former smoker (%)	1 (7%)
**Infection**	
Upper respiratory tract	4 (31%)
Urinary tract	2 (16%)
Cellulitis, endocarditis, cholecystitis	7 (54%)

**Table 5:** Characteristics at recruitment and after treatment where initial and final CRP levels were both > 100 mg/L.

Characteristic	Pre-treatment (mean ± SD)	Post-treatment (mean ± SD)	*Difference, 95% CI	*p-value
CRP (mg/L)	199.2 ± 59.0	159.4 ± 40.7	39.8 (− 1.0 to 80.6)	0.055
Central retinal arteriole equivalent calibre (MU)	147.7 ± 11.2	147.4 ± 3.3	0.3 (− 9.7 to 10.2)	0.96
Central retinal venular equivalent calibre (MU)	233.2 ± 27.0	235.0 ± 27.8	− 1.89 (− 16.1 to 12.3)	0.78

*CRP* C-reactive protein.

*Difference and P values are of paired results.

## Discussion

This study found that the retinal venular calibre in hospitalized patients with infections and CRP levels > 100 mg/L decreased when the follow-up CRP was < 100 mg/L after antibiotic treatment. There was no change in the arteriole calibre. There was also no change in calibre when individuals with infections were treated with antibiotics but their CRP levels did not fall below 100 mg/L. The reduction in venular calibre thus appeared to reflect the decrease in CRP level.

It is unlikely that the effect on venular calibre reflected the different types of infections or the antibiotics themselves since venular calibre did not change where the CRP level did not fall or when different antibiotics were used. It is also unlikely that smoking cessation in hospital or better blood pressure control was responsible for the change in calibre, because previous data suggest that the dilatation in smokers persists after they stop smoking^10^ and most patients' blood pressure measurements did not change during their treatment.

Population-based cohorts indicate that women with larger venules have an increased risk of cardiac events including stroke^9^, and coronary angiographic studies suggest that increased calibre correlates with cardiac events, coronary angiographic abnormalities and intracoronary plaque^2,3,25^. The reasons for these associations have been unclear. Diabetes, dyslipidemia and cigarette smoking are all traditional cardiac risk factors associated with venular dilatation, but venular dilatation is also associated with cardiac disease independent of these risk factors^26,27^. Non-traditional cardiac risk factors including obesity and rheumatoid arthritis also result in inflammation and venular dilatation^9,28^. Inflammation may have a direct effect on endothelial dysfunction and hence venular dilatation^29^. We are not arguing that transient infections predispose to macrovascular disease but rather that other sources of inflammation that are also cardiac risk factors such as diabetes and obesity may be responsible for the dilated venular calibre associated with cardiac events in large population-based studies. The transient venular dilatation that occurs with intercurrent infections may result in an erroneous assessment of high risk.

Retinal venular calibre reflects multiple systemic factors^8,29–32^ and is dynamic^12^. The initial white cell count in the cohort with infection was the only independent determinant of calibre identified after multivariate analysis. There was no association with CRP itself and the association with neutrophilia seen on univariate analysis did not persist. The observational nature of this study meant that it was not possible to exclude a shared cause for the increase in white cell counts and venular dilatation rather than the white cells directly affecting dilatation.  However a study from Rotterdam similarly found that a higher white cell count was associated with larger venular diameter^9^, and that this was explained by the infection-induced leucocytosis being partly modulated by  CRP level^33,34^.

Arteriole calibre was not altered in patients with active infections in this study. These results are consistent with previous findings that retinal venules, not arterioles, are dilated more in inflammation. Generally arteriole and venular calibre change in parallel, but arteriole calibre varies less, which may explain the lack of observable difference with infections noted here.

The strengths of this study were the careful characterization of participants, the highly reliable and reproducible method used for measuring microvascular calibre and the examination of individuals where the elevated CRP level persisted after antibiotic treatment. Although this cohort was smaller and younger, their calibre measurements still indicated that a lesser change in CRP was not associated with reduced venular calibre. The limitations of the study included that vessel calibre measurements from colour retinal images underestimate the vessel width because they measure the blood cell column rather than the peripheral plasma cuff that varies with the pulse cycle^30,35^.

It was unclear before this study was undertaken whether infections were associated with a change in retinal vessel calibre and indeed the amount of inflammation needed for any change. Very large population-based studies are required to detect a small change or to examine the effect of multiple covariates. This was a pilot study to determine the size of the effect, and a power calculation to determine the sample number was not possible. However similarly-sized studies examining venular calibre in inflammatory disease have also demonstrated a discernible change in calibre^11^.

It is unlikely that the decrease in venular calibre was due to intragrader variability because the retinal images were coded, and the grader was not aware of the nature of the study nor whether images were taken before or after antibiotic treatment. Although a number of potential participants were excluded because their CRP levels did not fall sufficiently or their images were not gradeable, there was no reason to believe that their clinical characteristics were the source of the different outcomes in calibre. The changes in calibre seen with infections lasted only a few days and were unaffected by other retinal structural parameters such as fractal dimensions or retinal nerve fibre layer thickness^36,37^.

The change in retinal venular calibre found here was small but may still be clinically relevant. Although some population-based studies exclude individuals with infections our results suggest that other kinds of inflammation associated with an increased CRP may affect calibre too, including coincidental gout, inflammatory arthritis, skin rashes, and surgery.

In summary, venular calibre may be affected reversibly in individuals with infections. Quantitative assessment of retinal microvascular calibre may be useful clinically in assessing blood pressure control or cardiac risk, or as a biomarker for risk stratification, for say, cardiac events. The present study demonstrates that hospital-based assessments of retinal microvascular calibre as a measure of blood pressure control or risk factor for cardiac disease must also consider coincidental infections and other sources of inflammation^11^ as potential confounders.

## Data Availability

The data is available in a deidentified format.
